# Small and Smaller—sRNAs and MicroRNAs in the Regulation of Toxin Gene Expression in Prokaryotic Cells: A Mini-Review

**DOI:** 10.3390/toxins9060181

**Published:** 2017-05-30

**Authors:** Sylwia Bloch, Alicja Węgrzyn, Grzegorz Węgrzyn, Bożena Nejman-Faleńczyk

**Affiliations:** 1 Department of Molecular Biology, University of Gdańsk, Wita Stwosza 59, 80-308 Gdańsk, Poland; sylwia.bloch@biol.ug.edu.pl (S.B.); grzegorz.wegrzyn@biol.ug.edu.pl (G.W.); 2 Institute of Biochemistry and Biophysics, Polish Academy of Sciences, Wita Stwosza 59, 80-308 Gdańsk, Poland; alicja.wegrzyn@biol.ug.edu.pl

**Keywords:** prokaryotes, non-coding small RNAs, microRNAs, toxin gene expression

## Abstract

Non-coding small RNAs (sRNAs) have been identified in the wide range of bacteria (also pathogenic species) and found to play an important role in the regulation of many processes, including toxin gene expression. The best characterized prokaryotic sRNAs regulate gene expression by base pairing with mRNA targets and fall into two broad classes: *cis*-encoded sRNAs (also called antisense RNA) and *trans*-acting sRNAs. Molecules from the second class are frequently considered as the most related to eukaryotic microRNAs. Interestingly, typical microRNA-size RNA molecules have also been reported in prokaryotic cells, although they have received little attention up to now. In this work we have collected information about all three types of small prokaryotic RNAs in the context of the regulation of toxin gene expression.

## 1. Introduction

The first hypothesis about the regulatory role of RNA molecules on the level of gene expression appeared in 1961 and was proposed by two outstanding scientists Francois Jacob and Jacques Monod [1]. This theory was quickly forgotten as many protein regulators were found in both prokaryotes and eukaryotes. Fortunately, it has aroused renewed interest in recent years. Over the last two decades an explosion in the study of RNA regulators, including non-coding small phage and bacterial RNAs (sRNAs), has been observed. Although a few hundred sRNAs have been predicted in the genome of *Escherichia coli*, one of the best-studied bacterial models, only about 100 have been experimentally validated up to now [2]. Interestingly, the majority of known *E. coli* sRNAs also occur in other, closely-related bacterial species. These molecules have been identified in a wide range of bacteria, including pathogens like *Salmonella* and *Yersinia*, and found to play an important role in the regulation of many processes such as carbon metabolism, virulence, motility, quorum sensing, biofilm formation, bacterial adaptation to changing conditions, and response to stresses [2,3,4,5,6]. The small RNAs occurring in prokaryotic cells vary in size between 50 and 500 nucleotide in length, show high structural diversity, and exhibit different molecular mechanisms of action. The most intensively studied prokaryotic sRNA regulators act by base pairing and share either extensive (*cis*-acting sRNAs) or more limited (*trans*-acting sRNAs) complementarity with their target transcripts [3].

In this work we gather information about two of the groups of small prokaryotic RNAs indicated above involved in the regulation of toxin gene expression. In addition, (for the first time in the review) we direct attention to microRNA-size molecules identified in prokaryotic cells, and present the outcome of the feature comparison between these and the above-mentioned *cis*-, and *trans*-acting sRNAs molecules (Table 1). We discuss the contribution of all these three types of sRNAs in the regulation of prokaryotic-cell based production of toxins. For transparency and clarity of the data presented in particular sections, we review separately sRNAs originating from the bacterial chromosome and molecules encoded within horizontally acquired genetic elements, with a particular focus on phage encoded sRNAs. In this report it was important to emphasize the significance of small RNAs molecules originating from phage genomes, as not enough attention has yet been given to this topic.

## 2. Prokaryotic Small RNAs (sRNAs) in the Context of the Regulation of Toxin Gene Expression

### 2.1. cis-Encoded sRNAs

**Figure 1:**
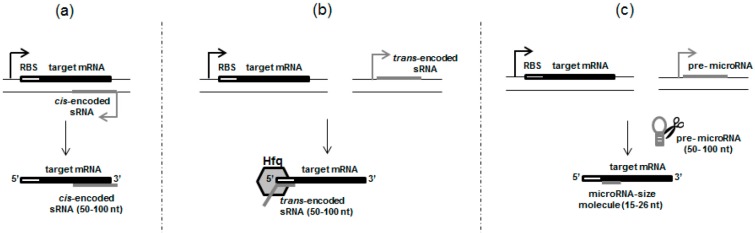
Schematic presentation of the three groups of small non-coding RNAs: (**a**) *cis*-encoded, (**b**) *trans*-encoded, and (**c**) microRNA-size molecules involved in the regulation of toxin gene expression in prokaryotic cells. Regulatory non-coding RNAs are shown in grey, whereas target mRNAs are shown in black. Size ranges of the respective types of non-coding RNAs are indicated in brackets and cover lengths most frequently reported in the literature.

The first group of small prokaryotic RNAs contains true antisense RNAs, which are synthesized from the complementary strand, at the same genetic locus as the target mRNA they regulate (Figure 1a). The overlap may occur at either the 5′ end or 3′ end of the mRNA transcript. It happens that the overlap includes the whole or fragments of the designated open reading frame [7]. The majority of *cis*-encoded sRNAs have broad secondary structures with very long stems and low numbers of unpaired nucleotides. In many cases these sRNAs have extensive complementarity (above 60 nucleotides) with their targets and pair rapidly. It helps to pair unstable RNAs and minimize the possibility of the RNAs’ dissociation. Usually, such an interaction does not require the presence of a chaperone like Hfq protein. However, additional protein factors might be involved to increase the specificity and efficiency of this regulation [8]. Antisense sRNAs are found mainly in phages and plasmids, although they also occur on bacterial chromosomes [7,8]. For clarity, some features essential for *cis*-acting sRNAs are summarized in Table 1, whereas examples of *cis*-acting sRNA-mediated regulations of toxin production are described below and listed in Table 2.

Most of the chromosomally, *cis*-encoded bacterial sRNAs have been reported to repress genes that encode small toxic proteins. Such an interaction has been classified in literature as the type I toxin-antitoxin (TA) pair [8]. The best known mechanism of the repression of toxic protein synthesis by antisense RNA assumes that sRNA base pairs across the ribosome binding site and blocks translation and/or provokes the mRNA degradation. Obviously, in some cases this regulation is more complex [8]. The Hok-Sok system of *E. coli* is one of the best characterized TA systems at present. This system is localized on the R1 plasmid, although the *E. coli* chromosome contains six homologous *hok*/*sok* loci. Additional homologs have also been found on the F plasmid (SrnB-SrnC) and R483 plasmid (PndA-PndB) [13]. Data have shown that a small antisense RNA (Sok) represses the synthesis of a small, hydrophobic protein (Hok) that kills the host cell by damaging the bacterial cell membrane, although it does not overlap the *hok* Shine-Dalgarno sequence. Instead, Sok RNA competes with a ribosome for the ribosome binding site of a neighbouring open reading frame *mok*, which almost entirely overlaps and is translationally coupled to that of the *hok*. As a result, Sok RNA indirectly inhibits the translation of *hok* mRNA by preventing the translation of *mok* [13]. Another type I toxin-antitoxin system has been identified on the *E. coli* chromosome within long direct repeat (LDR) sequences. This system includes protein toxin Ldr and RNA antitoxin Rdl. Overexpression of the small Ldr toxin leads to rapid host cell killing. In turn, *cis*-acting sRNA, named SymR is encoded opposite to the 5′ untranslated region of *symE* mRNA. SymE toxin synthesis might be induced during SOS response, and is strongly repressed at multiple levels including SymR RNA, which acts at the level of mRNA stability and translation [13,23]. Toxins regulated by sRNAs are likely to be integral membrane proteins. They are mostly hydrophobic, predicted to contain helical transmembrane domains and frequently similar to bacteriophage holin proteins. Even in small amounts they may cause damage to the bacterial membrane, and high levels lead to cell death. Other effects of protein toxins such as disruption of the nucleoid structure, chromosome segregation or cell division have also been observed. Antisense sRNA-based repression of toxin synthesis which occurs at the mRNA level and prevents production of toxic proteins, may have particular benefits. Contrary to antitoxin proteins which may dissociate from target toxins, the action of antitoxin sRNAs is usually irreversible as target mRNA is degraded. Besides, by preventing toxin production the cell reduces energy costs [8].

Among phage derived *cis*-acting sRNAs, one of the first described was the 77-nt molecule named OOP RNA, of bacteriophage λ [11]. This sRNA has been known for over 40 years and is encoded on the strand opposite to the strand of the *cII-O* transcript [24]. The OOP RNA-dependent cleavage of the cII mRNA was found to have physiological significance and to stimulate the lytic development of the λ bacteriophage [12,25]. Importantly, sequences similar to λ OOP RNA have been found in the *cII-O* regions of Shiga toxin-converting bacteriophages (Stx phages), responsible for the virulence of enterohaemorrhagic *E. coli* (EHEC) strains [12]. These pathogens cause serious food poisoning with bloody diarrhoea in humans. Their main virulence factors are Shiga toxins, encoded by genes *stx1* and *stx*2 located in the late region of the genome of the temperate bacteriophage, which occurs in bacteria as a prophage and is able to undergo both a lysogenic cycle and a lytic cycle. Lysogeny is one of the two phases of the temperate phage life cycle, characterized by the integration of phage nucleic acid into the bacterial genome to form a prophage, and by inhibition of the expression of the majority of phage genes. In such conditions bacteria grow and proliferate normally while phage DNA is replicating together with the bacterial genome and is transmitted to daughter cells during cell division. In this state, phage can be maintained for many cell generations. In the case of lamboid phages, to which Stx phages belong, the prophage state is achieved through the repression of the main lytic promoters, pL and pR, by binding of the phage cI repressor. As long as cI is active, the prophage is maintained, and production of majority of phage-derived proteins is suppressed. Many phage proteins, including Shiga toxins, whose genes are located on Stx prophages, are repressed in the lysogeny state, and their production occurs only after prophage induction. This step leads irreversibly to phage lytic development, host cell lysis and liberation of progeny virions [26,27]. The progressively produced Shiga toxins cause bloody diarrhoea—the first symptom of human infection [28,29]. The impact of Stx OOP RNAs on *stx* genes’ expression and thus production of Shiga toxins may be considered on the basis of their contribution in the regulation of the phage lytic cycle. Other examples of phage *cis*-encoded antisense RNAs have been found in virulent bacteriophage PAK_P3 during the early stage of infection of *Pseudomonas aeruginosa* bacteria. It was hypothesized that these antisense sRNAs may negatively impact the expression of late phage genes during the initial stage of infection, although this function has not been determined [30]. The presence of antisense RNAs expressed from the genome of bacteriophage φR1-37, infecting selected strains of *Yersinia enterocolitica,* has also been confirmed [31].

### 2.2. trans-Encoded sRNAs, Resembling Eukaryotic Small RNAs

The second group includes sRNAs, broadly described by Wagner and Romby [32], that also act by pairing, but have limited complementarity with their target mRNAs and are usually found at genomic locations remote from those of their targets (Figure 1b). In comparison with *cis*-acting sRNAs, they possess looser structures with shorter stems and higher numbers of unpaired nucleotides [3,8]. In addition, unlike *cis*-encoded sRNAs, small RNAs from this group usually have more than one mRNA target, and their pairing with targets frequently involves a highly-conserved seed region of 6–8 contiguous nucleotides (Table 1). For example, the widely conserved GcvB small RNA is able to regulate up to ~1% of all mRNAs in the Gram-negative bacteria species *Salmonella typhimurium* and *E. coli* [3,7,32]. In this light, *trans*-encoded RNA are often considered as the most related to eukaryotic microRNAs and siRNAs, and, similarly to them, they are able to regulate the translation and stability of target mRNAs. In addition to some common features, they differ in the details of their biogenesis and presentation on a protein scaffold. *Trans*-encoded bacterial sRNAs are generally transcribed as single transcripts, approximately 100 nucleotides in length and, unlike eukaryotic microRNAs and siRNAs, they are not processed to shorter fragments around 20-nt long. Interestingly, prokaryotic sRNAs derived by processing from longer RNAs also occur, although they are still longer than typical eukaryotic microRNAs and siRNAs [9,10]. Their transcription terminates frequently with a Rho-independent terminator, and the transcript is folded into a stable stem-loop structure which probably helps to keep stability. As indicated, *trans*-encoded sRNAs are more stable than their mRNA targets. In most cases these sRNAs, without further processing, bind to the Hfq chaperone which stabilizes sRNA and facilitates its base pairing with target mRNAs [7,33]. This protein is highly-conserved in prokaryotes. However, not all bacterial genomes contain the *hfq* homologue. Although the majority of *E. coli trans*-encoded sRNAs act by base pairing associated by the Hfq protein, some, such as sRNAs from other bacteria, e.g., *Vibrio cholerae, Listeria monocytogenes,* do not require this chaperone, even if Hfq is present in the host organism [34]. For comparison, the biogenesis of eukaryotic microRNA occurs through a multi-step process during which the primary microRNA (pri-microRNA) transcript, which can have from 10s to 100s of kilobases in length [35], undergoes subsequent enzymatic cuts to create a short microRNA duplex about 22 nucleotides in length. Next, one strand of this RNA duplex is incorporated into the RNA-induced silencing complex (RISC) where the mature microRNA interacts with its mRNA target [36]. Although both prokaryotic and eukaryotic small RNAs need protein cofactors which help in their presentation to the target mRNAs, the protein scaffold is more complex in the case of eukaryotic microRNAs. Another difference between bacterial *trans*-encoded sRNAs and the microRNAs of eukaryotes is the position of sRNA binding within the target mRNA transcript. In bacterial sRNAs, the overlap occurs frequently at the 5′ end of the target mRNAs, whereas eukaryotic microRNAs pair mainly at the 3′ untranslated region of the mRNAs. Base pairing between the prokaryotic *trans*-encoded sRNAs and mRNAs usually leads to mRNA degradation and/or translation blocking, but may also result in translation stimulation [33]. A brief overview of *trans*-encoded sRNA-based regulations of toxin production in prokaryotic cells is described below and presented in Table 2. 

One of the best known examples of *E. coli* sRNA-mediated regulation is the translation of the transcription factor RpoS (an alternative sigma factor of prokaryotic RNA polymerase), which is negatively influenced by the OxyS sRNA and positively regulated by at least two other small bacterial RNAs, DsrA, and RprA. Both of them pair with the region upstream of the translation start site and block the formation of a *cis*-inhibitory hairpin structure [37]. It was thought previously that the activation of translation by small RNAs is unique to prokaryotes [23]. However, as shown recently, eukaryotic microRNAs are also able to positively regulate translation [38,39]. Interestingly, *trans*-acting sRNAs (both positively and negatively impacted translation) have been recognized as essential factors involved in virulence regulation in many prokaryotic pathogens [2]. For instance, RNAIII, one of the largest known regulatory RNAs from *Staphylococcus aureus*, is a bifunctional molecule that encodes the δ-haemolysin protein in its 5′ end and additionally acts as a non-coding regulatory *trans*-encoded RNA. As indicated, this molecule can stimulate the translation of the staphylococcal alpha-toxin and regulate several other mRNAs with implications in virulence control [15,40]. Other interesting examples are *trans*-acting sRNAs named VR-RNA, VirX, VirU, and VirT encoded within the genome of *Clostridium perfringens*. These sRNAs are able to regulate the expression of various toxin genes, like *plc* (α toxin phospholipase C), *colA*, (κ toxin, collagenase), or *pfoA* (pore-forming toxin perfringolysin A), in different ways. However, their molecular mechanisms of gene regulation are still not understood [15,17,18]. Another molecule, FasX, was reported to have regulatory properties and to positively control the production of streptococcal haemolytic exotoxin streptolysin S (SLS) [15,16]. In some cases the antitoxin RNAs are also encoded in *trans*. One such example is identified in *E. coli* small RNA, named IstR-1. This molecule works as an antitoxin and inhibits the translation of the toxic protein TisB, which is an SOS-induced toxin that arrests cell growth under stress conditions [19].

A global study searching for small RNAs of phage origin was conducted by Tree and collaborators in 2014 [41]. They identified fifty-five Hfq-interacting sRNAs within phage-derived regions of the EHEC genome. Thirty-one of them were predicted to carry Rho-independent terminators. One of the best characterized molecules from this group is AgvB. This sRNA acts in a non-standard manner and mimics the mRNA target (DppA) of other small RNAs encoded in the bacterial chromosome and named GcvB. In detail, small RNA AgvB and DppA mRNA compete for binding to the Hfq protein and thereby also to GcvB. In this way AgvB antagonizes the functions of GcvB and, as indicated, aids the growth of Shiga toxin—producing *E. coli* bacteria within their animal host. Another small RNA, encoded by a prophage present in the genome of EHEC bacteria, has been identified by Sudo and collaborators [42]. This molecule is called Esr41, and was detected as an approximately 70-nucleotide long transcript with a predicted Rho-independent terminator and Hfq-interacting structure. Esr41 is classified as a *trans*-encoded sRNA and may regulate the expression of many genes from the bacterial motility regulation network, including *fliC*, which encodes the major subunit of flagella. Interestingly, the sequence of Esr41 is also present in *Shigella* bacteria [42]. Phage-derived small RNAs (~100 nt) have also been found at high levels in both a lysogen and during the late lytic cycle of mycobacteriophage, Giles [43]. Furthermore, acting in *trans* IsrK sRNA has been identified within the Gifsy-1 prophage of *Salmonella* bacteria. This molecule acts as small RNA to control the production of the toxic AntQ protein, playing a significant role in bacterial growth arrest and cell death [14]. *Trans*-encoded small RNAs have also been found in virulent bacteriophage PAK_P3, infecting pathogenic *Pseudomonas aeruginosa* bacteria. Interestingly, the identified small RNAs are strongly transcribed during late infection, but their role remains unclear [30]. An additional non-coding intragenic RNA molecule of unknown function has been identified within the genome of the φR1-37 bacteriophage infecting some strains of *Yersinia enterocolitica* [31]. Molecules selected from these two phages are the first examples of characterized small *trans*-acting RNAs encoded by virulent phages. Potential targets for these molecules have been found on the genomes of phage hosts which are human pathogens able to release dangerous toxins, although the functions of the identified molecules are not yet understood.

## 3. Something Smaller than sRNAs—True MicroRNAs in Prokaryotic Cells

The term “small molecules with great possibilities” fits the short, about 20-nucleotide RNA fragments, called microRNAs. Since the first such molecule was identified over 20 years ago in 1993 [44], a growing number of microRNAs have been found in humans, plants, and animals. Up to 2014, 24,521 microRNAs had been discovered, and they are important components of many regulatory pathways [45]. Although such small molecules have been found broadly in multicellular organisms, there is an opinion that typical prokaryotic microRNAs do not occur [46]. Interestingly, a few microRNA-size small RNA fragments (15–26 nt) have recently been reported in prokaryotic cells. Such prokaryotic RNAs of comparable size to eukaryotic microRNAs have received little attention up to now and, therefore, they are presented in this section, summarized in Table 1 and schematically illustrated in Figure 1c. 

So far, microRNA-size RNA fragments have been identified in a few different species of bacteria. Such molecules were found in *Streptococcus mutans* in 2011 [47] and in *E. coli* in 2013 [48], although their functions remain to be determined. Another microRNA of bacterial origin has been identified in *Mycobacterium marinum*, a fish pathogen able to produce a unique mycolactone toxin, mycolactone F [21]. This 23-nt small RNA molecule, named MM-H, is derived from a precursor stem-loop structure, requires the mammalian host cell RNA processing machinery for its biogenesis, and probably also affects the expression of the eukaryotic host, rather than bacterial genes [21]. Small RNAs comparable in size to eukaryotic microRNAs have also been identified in periodontal pathogens such as: leukotoxin LtxA—producing *Aggregatibacter actinomycetemcomitans*; engaged in the production of the haemolytic toxin *Porphyromonas gingivalis* strain; responsible for production of highly toxic metabolites *Treponema denticola* bacteria [22] and a key player in the colonization of the human oral cavity *Streptococcus sanguinis* [49]. It was observed that the identified microRNA-size RNAs are secreted from the periodontal pathogens via bacterial outer membrane vesicles (OMVs) and affect the eukaryotic host immune system by suppressing the expression of certain cytokines [22]. Interestingly, OMVs serve as secretory vehicles by which pathogens not only modulate the host immune response, but also deliver toxins into host cells and are, thus, considered as a potent virulence mechanism [50]. In this light, the identified microRNA-size molecules may function as novel bacterial signalling molecules that mediate both the bacteria-eukaryotic host and bacteria-bacteria interactions during infection [21,22,49]. 

Our group presented the phage-derived microRNA-size (20-nt long) molecule which had been isolated from *E. coli* culture after the induction of Shiga toxin-converting bacteriophage Φ24B, which is responsible for the virulence of EHEC strains [20]. This small RNA, named 24B_1, is encoded in the *lom-vb_24B_43* region of the phage Φ24B genome. Apparently, it is produced by the cleavage of a larger (80-nt long) transcript, which was also detected. Since it appeared quite abundant in the next-generation sequencing analysis, we assume that 24B_1 is a product of the specific cleavage of the longer transcript [20]. Additionally, using Mfold web server: 1995–2017 (hosted by the RNA Institute, College of Arts and Sciences, State University of New York at Albany, NY, USA) we were able to predict the hairpin RNA structure of the identified 80-nt transcript. As both the secondary structure of the precursor and its cleavage to the shorter form resemble the formation of microRNAs in eukaryotic cells [51], we suggest that 24B_1 might be formally considered as this type of sRNA. Additionally, in silico analyses showed the presence of two potential binding sites for this molecule within the phage genome. Interestingly, 24B_1 displays limited complementarity with both of the identified targets. Apart from that, we have demonstrated that this small molecule has a physiological role and promotes phage lysogeny—a part of the phage life cycle in which most of the phage genes are not expressed and proteins are not produced. Such a conclusion is supported by results showing that the mutant phage lacking the sequence encoding the whole precursor revealed dramatic changes in the expression of all tested phage genes during *E. coli* infection, and significant differences in various phage developmental processes [20]. As we know, 24B_1 is the first phage–derived microRNA-size RNA molecule identified so far, and this is the first demonstration of the physiological significance of a phage microRNA-size molecule in bacterial cells [20]. 

All the above-presented prokaryotic microRNA-size molecules [20,21,22,47,48,49] have been identified by next generation sequencing (NGS). Such an experimental approach and bioinformatics analysis allowed for extracting large amounts of data and the identification of, e.g., more than 400 [48] or more than 900 [47] individual RNAs of microRNA size (15–26 nt) in a single experiment. Up to now, the cellular abundance of about 30 molecules identified by NGS [20,21,22,47,48,49] was validated experimentally, mainly by qRT-PCR and/or Northern blot techniques. Furthermore, in silico analyses allowed for the prediction and presentation of a total of 24 hairpin-structured precursors for the validated prokaryotic microRNA-size molecules [20,21,22,47,48,49]. Within that group, the expression of seven longer, potential precursor transcripts, covering the predicted microRNAs loci, have been confirmed in bacterial cells by the RT-PCR method [20,22]. The exact cleavage site of the identified hairpin precursors are not yet confirmed. However, the presence of single, unsmeared bands of analysed microRNAs revealed by Northern blot [47,49] and the observation that they occur in NGS data in only one fixed-length form [20] suggest that at least some of the found molecules may be a result of the specific cut of the longer precursor. In the light of the current knowledge that the presence of a longer hairpin precursor is crucial for the final processing of eukaryotic microRNAs [52], we suggest that at least seven molecules discussed here might be considered as microRNA type molecules [20,22]. On the basis of the available knowledge, we cannot exclude that, similarly to eukaryotic microRNAs, prokaryotic microRNAs undergo a multi-step maturation process. As a reminder, in the first step of microRNA biogenesis in eukaryotic cells, RNA polymerase generates a very long pri-microRNA transcript that can extend hundreds of kilobases in length. This pri-microRNA undergoes nuclear cleavage, by the protein complex containing the Drosha enzyme, to form a short, about 70-nt, hairpin structure known as precursor microRNA (pre-microRNA). This precursor RNA is then exported from the nucleus by Exportin-5. In the cytoplasm, the pre-microRNA is processed by Dicer enzyme into a mature molecule of about 22-nt miRNAs. As the Dicer enzyme seems to be essential for the maturation of eukaryotic microRNAs, it was suggested that the same role may be served by the MazF enzyme in *E. coli* bacteria [47]. Importantly, alternative Drosha- and Dicer-independent microRNA biogenesis pathways have also been proposed in eukaryotes [53]. In the search for other similarities between eukaryotic and prokaryotic microRNA-size molecules, attention has been attracted to the proposed mechanism of microRNA transfer by OMVs [22,49]. Studies of bacterial OMVs have revealed some similarities with exosomes involved in eukaryotic microRNAs transport [54]. It was proven that, similarly to exosomal microRNAs, prokaryotic microRNA-size molecules found in OMVs are stable and well protected from degradation by ribonucleases [22,49]. Additionally, by analogy to exosomal microRNAs, it is possible to suggest a probable role of prokaryotic microRNAs as signalling molecules involved in communication among prokaryotic cells and/or between prokaryotic and eukaryotic cells. Importantly, such a signalling function can also be considered in the light of scientific data showing that identified microRNAs may regulate the phage lifecycle [20], may suppress the expression of certain cytokines [22], and may be involved in vitamin B12 conversion [49]. 

The question emerges of whether the identified microRNAs should be seen in the context of the regulation of prokaryotic-cell based toxin production. It is difficult to answer this question clearly now, as very little information is known about their function in prokaryotic cells. Nevertheless, it seems likely—or at least it cannot be excluded, as some provided examples of microRNAs might be considered in this context (Table 2). Our investigation has shown that microRNA-size molecule 24B_1 may act as a negative regulator of the expression of the *d_ant* gene which encodes an antirepressor of the phage cI repressor, and thus indirectly stimulates the phage lysogenic state [20]. As cI is responsible for maintaining the lysogenic life cycle, its deactivation results in prophage induction, production of phage proteins (including toxins), and release of progeny phages. In the light of the ability of the identified microRNA size 24B_1 molecule to control phage lysogeny, we may also consider this molecule in the context of the regulation of Shiga toxins gene expression. It should, however, be emphasized that the direct regulation of the expression of Shiga toxins genes by 24B_1 was not proven, and that, to our knowledge, this is the first and only example of phage microRNA-dependent regulation of toxin gene expression in bacterial cells [20]. Other identified examples of such regulation and further research are required to confirm that this is a widespread phenomenon. However, in the frame of this work we wanted to attract attention to this issue. This seems to be particularly important in view of the well-recognized role of bacteriophages as vectors of bacterial toxins [55]. Several different types of bacterial virulence factors, including not only Shiga, but also diphtheria, botulinum, cholera, and other toxins, are encoded on a diversity of lysogenic phages, frequently tailed dsDNA phages [56]. In the light of the foregoing, we cannot exclude that other phage or bacterial origin microRNA-size molecules affecting the regulation of toxin gene expression may be identified in the future. Bearing in mind that eukaryotic microRNAs can regulate tens to hundreds of protein-coding genes, and a single transcript can be regulated by several microRNAs of the same or different sequence [57,58], we can speculate that microRNAs in prokaryotic cells present similar abilities, and would also be able to regulate multiple targets. If this is true, many biological processes, including toxins production, may be largely influenced by microRNAs occurring in prokaryotic cells.

## 4. Conclusions

Many small non-coding RNAs have been identified as crucial regulatory elements in the virulence of bacteria. A major class of prokaryotic sRNAs act by pairing with extensive or more limited complementarity with their target transcripts. *Trans*-encoded sRNAs are the largest class of prokaryotic non-coding RNAs, and for long time they have been considered as the equivalents of the eukaryotic microRNAs and siRNAs. Although they resemble eukaryotic small RNAs in their biological effects, they vary considerably in the details of their synthesis and presentation on a protein scaffold to the target mRNAs [33]. The view that *trans*-acting sRNAs are the most related to eukaryotic small RNAs is changing slightly since the true prokaryotic microRNAs have been reported [20,21,22,47,48,49]. Unquestionably, microRNAs identified so far in prokaryotic cells serve as the starting points for further studies in this new, largely unexplored research area. In comparison to the very large number of reports concerning eukaryotic microRNAs, current knowledge about such microRNAs in prokaryotic systems is only ‘a drop in the ocean’ and there is an urgent need to continue research in this field.

## Figures and Tables

**Table 1:** Comparison of the main features of three types of prokaryotic non-coding sRNAs.

Feature	*cis*-Acting sRNAs	*trans*-Encoded sRNAs	MicroRNA-Sized Molecules
Action on the target gene Complementarity with targets One or multiple targets Accompanying proteins Structure Processing	*cis*-regulation extensive one usually not required broad secondary structures with very long stems does not occur	*trans*-regulation limited multiple usually required more loosely structures with shorter stems usually does not occur ^2^	*trans*-regulation limited multiple ^1^ not determined usually single stem-loop precursor structure occur ^3^
The most common size range of the mature sRNA molecule (nt)	50–100	50–100	15–26

^1^ Multiple targets have been predicted but not confirmed experimentally; ^2^ In a few cases processing from longer RNA to a shorter fragment occurs [9,10]. However, mature molecules are still longer than typical, approximately 20-nucleotide microRNAs; ^3^ The mechanism of processing from precursors to shorter forms has not yet been determined.

**Table 2:** Examples of *cis*-acting, *trans*-encoded and microRNA-size non-coding RNA molecules involved in the regulation of toxin production and secretion by prokaryotic cells.

sRNA Type	sRNA Name	sRNA Source	sRNA Function in the Context of the Regulation of Toxin Production and Secretion	Ref.
*cis*-encoded sRNAs	OOP	Bacteriophage λ	Is predicted to repress the synthesis of the cII protein and thus indirectly regulate the production of Shiga toxins by Stx phages	[11,12]
	Sok	Plasmid R1 & chromosome of *E. coli*	Indirectly inhibits the synthesis of the highly toxic protein Hok, responsible for cell membrane damage	[13]
	Rdl	Chromosome of *E. coli*	Regulates the synthesis of the Ldr toxin, whose over expression leads to rapid host cell killing	[13]
	SymR	Chromosome of *E. coli*	Regulates the endogenous level of the SymE toxin	[13]
*trans*-encoded sRNAs	IsrK	Bacteriophage Gifsy-1	Controls the production of the toxic AntQ protein which is responsible for bacterial growth arrest and cell death	[14]
	RNAIII	Chromosome of *Staphylococcus aureus*	Induces the expression of genes encoding the staphylococcal alpha-toxin	[15]
	FasX	Chromosome of *Streptococcus pyogenes*	Positively controls the production of streptococcal haemolytic exotoxin streptolysin S (SLS)	[16]
	VR-RNA	Chromosome of *Clostridium perfringens*	Is responsible for the regulation of the expression of toxin genes, such as *plc* (α toxin phospholipase C) and *colA*, (κ toxin, collagenase)	[17]
	VirX	Chromosome of *Clostridium perfringens*	Regulates the expression of genes *: plc*, *colA* and *pfoA* coding for pore-forming toxin perfringolysin A; Controls the production of enterotoxin	[18]
	VirU	Chromosome of *Clostridium perfringens*	Has a positive effect on the production of pore-forming toxin perfringolysin A	[18]
	VirT	Chromosome of *Clostridium perfringens*	Negatively regulates *pfoA* and *colA* transcription	[18]
	IstR-1	Chromosome of *E. coli*	Inhibits translation of the toxic protein TisB, which is responsible for cell growth arrest under stress conditions	[19]
microRNA-size RNAs	24B_1	Bacteriophage Φ24B	Is predicted to regulate the phage *d_ant* gene, and thus indirectly stimulate the lysogenic state of the Stx phage during which Shiga toxins are not produced	[20]
	MM-H	Chromosome of *Mycobacterium marinum*	Does not have a defined function	[21]
	AA_20050 PG_122 TD_16563	Chromosome of *Aggregatibacter actinomycetemcomitans*, *Porphyromonas gingivalis*, *Treponema denticola*	Do not have defined functions and are transmitted by outer membrane vesicles (OMVs) that enable bacteria to secrete a large, complex group of proteins, including toxins	[22]
